# Comments on “Effects of refractive accommodation on subfoveal choroidal thickness in silicone oil-filled eyes” BMC Ophthalmology (2022) 22:107 https://doi.org/10.1186/s12886-022-02332-y

**DOI:** 10.1186/s12886-022-02696-1

**Published:** 2022-12-08

**Authors:** Lei Gao, Yang Zhou, Guangsen Liu

**Affiliations:** 1 Department of Ophthalmology, Weifang Eye Hospital, Zhengda Guangming Eye Group, 139 Xingfujie, Weifang, 261000 Shandong China; 2 Zhengda Guangming International Eye Reserch Center, Qingdao University, Qingdao, China

**Keywords:** Accommodation, Silicone oil, Ocular parameters, Choroidal thickness

Dear Editor:

When we searched the literature on accommodation, we occasionally found an article entitled “Effects of refractive accommodation on subfoveal choroidal thickness in silicone oil-filled eyes” published in BMC Ophthalmology (2022) 22:107, which aroused our curiosity as doctors engaged in vitreoretinal specialties.

As we all know, the influence of accommodation on eye parameters is still worth further exploring, such as anterior chamber depth, vitreous cavity length and axial length may change when accommodation was initiated [1]. However, it is difficult to imagine that accommodation can affect choroidal thickness in silicone oil-filled eyes within 24 h, according to the existing Helmholtz theory of accommodation [2, 3].

If accommodation can affect choroidal thickness in silicone oil-filled eyes, that is, even in non-silicone oil-filled eyes, accommodation can theoretically affect the choroidal thickness. This is because accommodation can occur anytime and anywhere, regardless of whether silicone oil is used. Silicone oil, as a tamponade, is colorless, tasteless, non-toxic, and non-flexible.

In this study, the effect of accommodation on choroidal thickness in silicone oil-filled eyes was determined indirectly. A more scientific conclusion should be the short-term effect of the hyperopia contact lens on choroidal thickness in silicone oil-filled eyes. The possible changes that occur after wearing a hyperopic contact lens should be analyzed. However, based on our current knowledge of the Helmholtz theory of accommodation, it is difficult to imagine that wearing contact lenses to correct hyperopia has a significant effect on choroidal thickness.

We fully agree with the authors’ view that the choroidal thickness was quite different for hyperopic and myopic patients. However, the difference in choroidal thickness is due to long-term effects of the refractive state. Another important fact is that, regardless of myopia or hyperopia, the most significant difference between the two refractive states is the difference in the axis length, and the axis length of the eye is undoubtedly more likely to lead to changes in choroidal thickness [4, 5].

There are still some problems in this study:1) The average age of the study was 38 years old, and the amplitude of accommodation was only about 4.5D, which is much lower than the 14D of 10-year-old children, so it is doubtful that such an amplitude of accommodation can produce a significant effect on choroidal thickness within 24 h. If the effect of accommodation on choroidal thickness is to be studied, a population of children who are eligible for OCT should be the first choice, as their amplitude of accommodation is much greater than that of adult; 2) the refractive state of the contralateral eye and the best-corrected visual acuity were not provided. According to the theory of accommodation, the accommodation used by both eyes is symmetrical and equal and often takes the dominant eye as the reference [1]. Considering that the control group was a healthy eye, and the experimental group was patients only 1 month after the operation, the patient took the healthy eye as the dominant eye. In other words, even if the surgical eye wears a hyperopic contact lens, the accommodation induced by the contact lenses may also be negligible. 3) According to the existing accommodation theory, the realization of accommodation mainly depends on the change in lens refractive power, and the change in lens refractive power in a short time is mainly reflected in the changes in lens configuration or more directly and easily observed changes in lens thickness. However, this study obviously does not provide the difference in lens thickness before and after wearing contact lenses.

In summary, although there are no obvious flaws in the study design, according to the existing and recognized ophthalmic knowledge system, it is inconceivable that a contact lens can significantly affect the choroidal thickness of silicone oil-filled eyes within 24 h unless there are other theoretical breakthroughs. We recommend that in addition to increasing the sample size, automatic data acquisition be used as much as possible to minimize the bias caused by manual measurement.

## Data Availability

Not applicable.
